# Phospho-Ablated Id2 Is Growth Suppressive and Pro-Apoptotic in Proliferating Myoblasts

**DOI:** 10.1371/journal.pone.0006302

**Published:** 2009-07-17

**Authors:** David C. Butler, Satoshi Haramizu, David L. Williamson, Stephen E. Alway

**Affiliations:** Laboratory of Muscle Biology and Sarcopenia, Department of Exercise Physiology, West Virginia University School of Medicine, Morgantown, West Virginia, United States of America; Roswell Park Cancer Institute, United States of America

## Abstract

Inhibitor of differentiation protein-2 (Id2) is a dominant negative helix-loop-helix (HLH) protein, and a positive regulator of proliferation, in various cells. The N-terminal region of Id2 contains a consensus cdk2 phosphorylation sequence SPVR, which may be involved with the induction of apoptosis, at least in myeloid 32d.3 cells. However, the role of Id2 phosphorylation at serine 5 in skeletal muscle cells is unknown. The objective of this study was to determine if the phosphorylation of Id2 at serine 5 alters its cellular localization and its role in apoptosis in C2C12 myoblasts. Overexpression of wild type Id2 decreased MyoD protein expression, which corresponded to the increased binding of Id2 to basic HLH proteins E47 and E12. Bromodeoxyuridine incorporation was significantly decreased by the overexpression of phospho-ablated Id2 (S5A); conversely, overexpression of wild type Id2 increased cellular proliferation. The subcellular localization of Id2 and phospho-mimicking Id2 (S5D) were predominantly nuclear compared to S5A. The decreased nuclear localization of S5A corresponded to a decrease in cellular proliferation, and an increase in apoptosis. These data suggest that unphosphorylated Id2 is primarily localized in the cytosol, where it is growth suppressive and potentially pro-apoptotic. These results imply that reducing unphosphorylated Id2 may improve the pool of myoblasts available for differentiation by increasing proliferation and inhibiting apoptosis.

## Introduction

Myogenesis requires that muscle precursor cells (e.g. satellite cells), or myoblasts, undergo proliferation, followed by cell-cycle exit. This is followed by myogenic differentiation, and finally cell fusion into multinucleated myotubes then myofibers. Skeletal muscle growth and regeneration/repair are critically dependent on having an adequate pool of muscle precursor cells or myoblasts. Consequently loss of myoblasts during the initial stages of myogenesis limit or prevent muscle growth or in aging, may contribute to sarcopenia by reducing the ability to replace muscle mass during normal protein turnover [1]. Therefore, the identification and characterization of important proteins that regulate the expansion and survival of myoblasts are important for both increasing our understanding of myogenesis, but also for identifying strategies that will reduce the loss of muscle mass that occurs with aging and disease.

While the majority of muscle precursor cells/myoblasts exit the cell cycle and undergo terminal differentiation during myogenesis and muscle repair/growth, ∼30% of differentiating myoblasts undergoes cell death during differentiation [2], [3]. Interestingly, signaling pathways required for the initiation and execution of programmed cell death, or apoptosis, are activated during myogenesis [4], [5]. For example, caspase 3 is not only activated during myogenesis, but also its activity is required for the initiation of myogenic differentiation [6], [7]. The mechanism by which the majority of muscle cells undergo caspase-dependent differentiation but escapes caspase-dependent or caspase-independent apoptosis is not clear.

Control of apoptosis in myoblast proliferation and differentiation is critical for development and muscle repair/growth. We propose that inhibitor of differentiation (Id) protein may play roles in both regulating proliferation/differentiation and apoptosis in skeletal muscle cells. The Id family consists of helix-loop-helix proteins (HLH) that act as negative regulators of cell differentiation in many cell types including skeletal muscle [8]–[11]. Id proteins were identified because of their sequence homology to the second amphipathic helix shared by other basic HLH (bHLH) proteins such as MyoD, E47, and E12 [12]. Homodimers and heterodimers of bHLH proteins regulate the transcription of targeted genes such as muscle creatine kinase (MCK) by binding to a palindromic E-box motif, CANNTG [9], [12], [13]. Id proteins lack the basic N-terminal region needed for DNA binding and inhibit myogenic differentiation by sequestering bHLH proteins E47 and E12.

The levels of Id proteins are high during proliferation and decrease prior to the onset of terminal differentiation in many cell types [12], [14], [15]. Enforced overexpression of Id genes has been shown to suppress myogenic differentiation, and the expression of Id genes is rapidly increased when quiescent cells are stimulated with serum [10], [15].

Four members of the Id family have been identified (Id1 to Id4). Id2, Id3, and Id4 share a conserved amino acid sequence SPVR, which is a phosphorylation target of cyclin E-cdk2 and cyclin A-cdk2 kinases. Id2 phosphorylation on serine 5 by cyclin A/cdk2 has been shown to restore E12/E12 and E12/MyoD binding to DNA [15]. Interestingly, phospho-ablated Id2 mutants where serine 5 has been mutated to an alanine are growth inhibitory in fibroblasts, smooth muscle cells and osteosarcoma cell lines [15], [16], although it is not known if this is the case in myoblasts.

Ectopic overexpression of Id2 promotes S-phase entry in smooth muscle cells [16]. Conversely, treatment with antisense oligonucleotides complementary to Id2 inhibits cell cycle progression in serum stimulated fibroblasts [15]. In human fibroblasts and neuroblastoma cell lines, Id2 promotes G_1_-S progression by binding to the active, hypo-phosphorylated, form of retinoblastoma protein pRb [17]–[19]. Rb family proteins p107 and p130 are also antagonized by Id2 [20].

Id2 overexpression has also been linked to apoptosis in various cell lines [8]. In 32D.3 myeloid progenitor cells, the N-terminal region of Id2 is able to induce apoptosis independent of its HLH function [21]. In aged rodent skeletal muscle, where Id2 levels are elevated, there is a positive correlation between Id2 and pro-apoptotic proteins Bax and caspase 9 [22].

Previous data show important roles for apoptosis in skeletal muscle adaptations including loss of skeletal muscle during aging [23]–[25]. We have previously speculated that the subcellular localization of Id2 may be important in regulating apoptosis in skeletal muscle [26], [27]. Id2 cytoplasmic levels are elevated during conditions of atrophy in skeletal muscle, and positively correlated to proteins associated with the intrinsic pathway of cell death [26], [27]. Phospho-ablated mutants of Id2 at serine 5 are primarily localized to the cytoplasm in primary rat aortic smooth muscle cells [16], although it is not known if this is also the case in skeletal muscle cells. Furthermore, the role of Id2 phosphorylation at serine 5 is unknown in skeletal muscle and myogenic cell lines. In this study we tested the hypothesis that Id2 phosphorylation at serine 5 regulates apoptosis and cell cycle progression in myoblasts.

## Results

### Phospho-ablated Id2 inhibits G1/S transition

**Figure 1:**
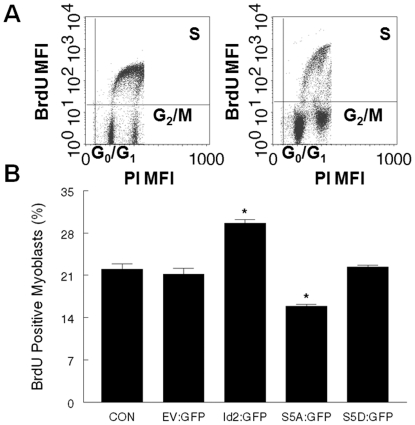
Phospho-ablated Id2 reduces BrdU incorporation in myoblasts. Twenty four hours after transfection, the myoblasts were pulsed with BrdU, and subjected to flow cytometric analysis. (A) Representative dot plot of Id2:GFP (left) and S5A:GFP (right). *Y-axis* = BrdU-Alexa 488 MFI. *X-axis* = FL2-Area. The upper right quadrant contains cells that are S-phase/BrdU positive. G_0_/G_1_ and G_2_/M cell populations are determined by MFI of 7AAD intercalating into the DNA. (B) Summary of cell cycle data. [*, Id2:GFP and S5A:GFP are significantly different from all groups (p<0.05)].

The manner in which phosphorylated and phospho-ablated Id2 affected myoblast proliferation was measured by bromodeoxyuridine (BrdU) incorporation into S-phase of the cell cycle (Figure 1A). S-phase myoblasts were determined from flow cytometric analysis of BrdU expression (y-axis) and DNA content (x-axis). Overexpression of Id2:GFP resulted in a significant increase in the percentage of BrdU positive cells (Figure 1B) compared to all other groups. Conversely, overexpression of S5A:GFP resulted in a significant decrease in BrdU positive cells.

### Phospho-ablated Id2 is pro-apoptotic in C2C12 myoblasts

**Figure 2:**
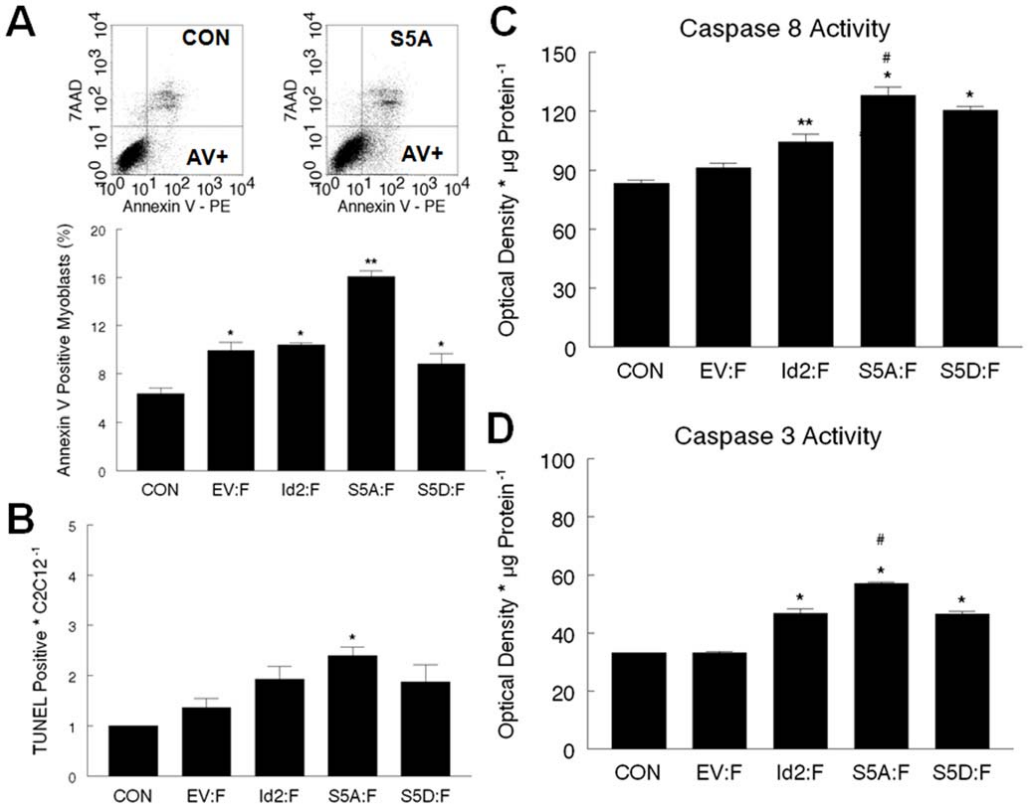
Overexpression of phospho-ablated Id2 is pro-apoptotic. Control C2C12 myoblasts (CON) were transfected with, EV:F, Id2:F, S5A:F, and S5D:F. Twenty-four hours after transfection, cells were assayed for apoptosis. (A) Summary of Annexin V data. [*, significantly different from CON (p<0.05): #, significantly different from all groups (p<0.05).] Insert: Representative dot plots. (*y-axis* = 7AAD, *x-axis* = AnnexinV PE). Cells in the lower left quadrant are viable and 7AAD negative. Cells in the upper right quadrant were considered to be necrotic, and are annexin V positive and 7AAD positive. Cells in the lower right quadrant are apoptotic, and Annexin V positive. (B) There is a significant increase (p<0.05) in the percent of cells TUNEL positive in C2C12 myoblasts overexpressing phospho-ablated S5A:F than in all other groups. (C) Caspase 8 activity. [*, significantly different from CON, EV:F (p<0.05) **, significantly different from CON (p<0.05) #, significantly different from Id2:F(p<0.05)]. (D) Caspase 3 activity assay. [*, Significantly different from CON and EV (p<0.05): #, significantly different from all groups (p<0.05)].

To determine if the apoptotic role of Id2 is determined by the phosphorylation status of this protein, C2C12 myoblasts were transfected with Id2:GFP, S5A:GFP, or S5D:GFP. The cells were harvested 24 hours after transfection and analyzed for Annexin V expression (Figure 2). The cells' surface expression of phosphatidylserine is a hallmark of cellular death associated with apoptosis [28]. Viable cells are Annexin V negative and 7-amino-actinomycin D (7AAD) negative. Cells in the early phases of cell death are Annexin V positive and 7AAD-negative, and necrotic cells are Annexin V positive and 7AAD-positive (Figure 2A). Although transfection per se did result in a low level of apoptosis as shown by an elevation in Annexin V positive cells (Figure 2A), a less sensitive TdT-mediated dUTP nick end labeling (TUNEL) assay, which measures the free 3′OH end of DNA, did not show an increase in the frequency of apoptotic cells in the vector-only group (Figure 2B). In contrast, overexpression of phospho-ablated Id2 (i.e., S5A) resulted in a significant increase in the percentage of Annexin V positive myoblasts (Figure 2A) as well as an increase in the percentage of TUNEL positive myoblasts (Figure 2B). Because other processes such as necrosis and DNA repair result in free 3′OH production, caspase 3 and caspase 8 activities were measured to confirm apoptotic signaling in Id2S5A transfected cells. Overexpression of Id2:F, S5A:F, and S5D:F resulted in a significant increase of caspase 3 activity compared to CON and EV:F (Figure 2D); however, caspase 3 activity was significantly higher in S5A:F transfected myoblasts than all other groups. Overexpression of S5A:F resulted in an increase of caspase 8 activity (Figure 2C) compared to CON, EV:F, and Id2:F transfected myoblasts.

### Wild-type Id2, phospho-ablated Id2 (S5A), and phospho-mimicking Id2 (S5D) successfully bind to E47/E12

**Figure 3:**
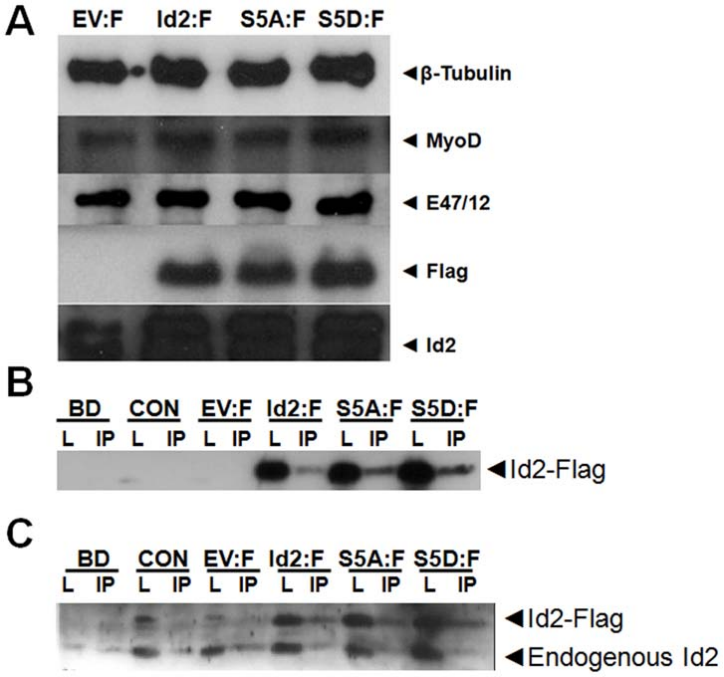
Id2-3Xflag fusion proteins bind to E-proteins. (A) MyoD and E47 protein levels are not affected by the overexpression of Id2:F, S5A:F or S5D:F compared to control EV:F in whole cell lysates. An anti-E12/E47 IP was performed with 500 µg of whole-cell lysates and anti-E47/E12 antibodies. The precipitates were blotted with anti-M2 flag monoclonal antibodies (B), and anti-Id2 polyclonal antibody (C). BD = bead only control; CON = control C2C12; EV:F = Flag Empty Vector; Id2:F = Id2-Flag; S5A:F = Id2S5A-Flag; S5D:F = Id2S5D-Flag.

Id2-3XFlag fusion proteins were generated as a means for identifying exogenous Id2 from endogenous Id2. To confirm that flag positive myoblasts were also Id2 positive, transfected myoblasts were double labeled for Id2 and flag expression (Supplement Figure S1). As expected, flag positive myoblasts were also Id2 positive. There was approximately a 15 fold increase of Id2 MFI in Id2:F, S5A:F, and S5D:F transfected myoblasts compared to control (CON) and empty flag vector (EV:F) controls (Supplement Figure S1). Id2 has been shown to preferentially bind to the ubiquitously expressed E-proteins compared to tissue restricted class B bHLH proteins such as MyoD [29]. Western blots analysis were performed to determine if the overexpression of Id2-3XFlag fusion proteins alter the protein expression of bHLH proteins. The protein expression of MyoD and E47/E12 from whole cell lysates was not altered by the overexpression of Id2:F, S5A:F, and S5D:F compared to control cell lysates. Immunoprecipitation of anti-E47/E12 was performed to determine if the phosphorylation of Id2 at serine 5 affects the ability of Id2 to dimerize with E47/E12. The precipitates were probed with anti-M2 flag antibody and there was a single band present at the 18 kDa molecular weight marker (Figure 3B). To confirm endogenous Id2 binding to E-proteins, the E47/E12 precipitates were probed with anti-Id2. As expected, endogenous Id2 was bound to E47/E12 in CON and EV groups (Figure 3C). There were non-specific bands migrating at 18 kDa in the lysates of CON and EV:F; however, positive bands were not located in the corresponding precipitates. Id2:F, S5A:F, and S5D:F groups had bands migrating at 18 kDa, which correlated to the Id2 fusion proteins. MyoD was not detected in any of the E47/E12 precipitates (data not shown).

### Phospho-ablated Id2 is primarily localized in the cytosol

**Figure 4:**
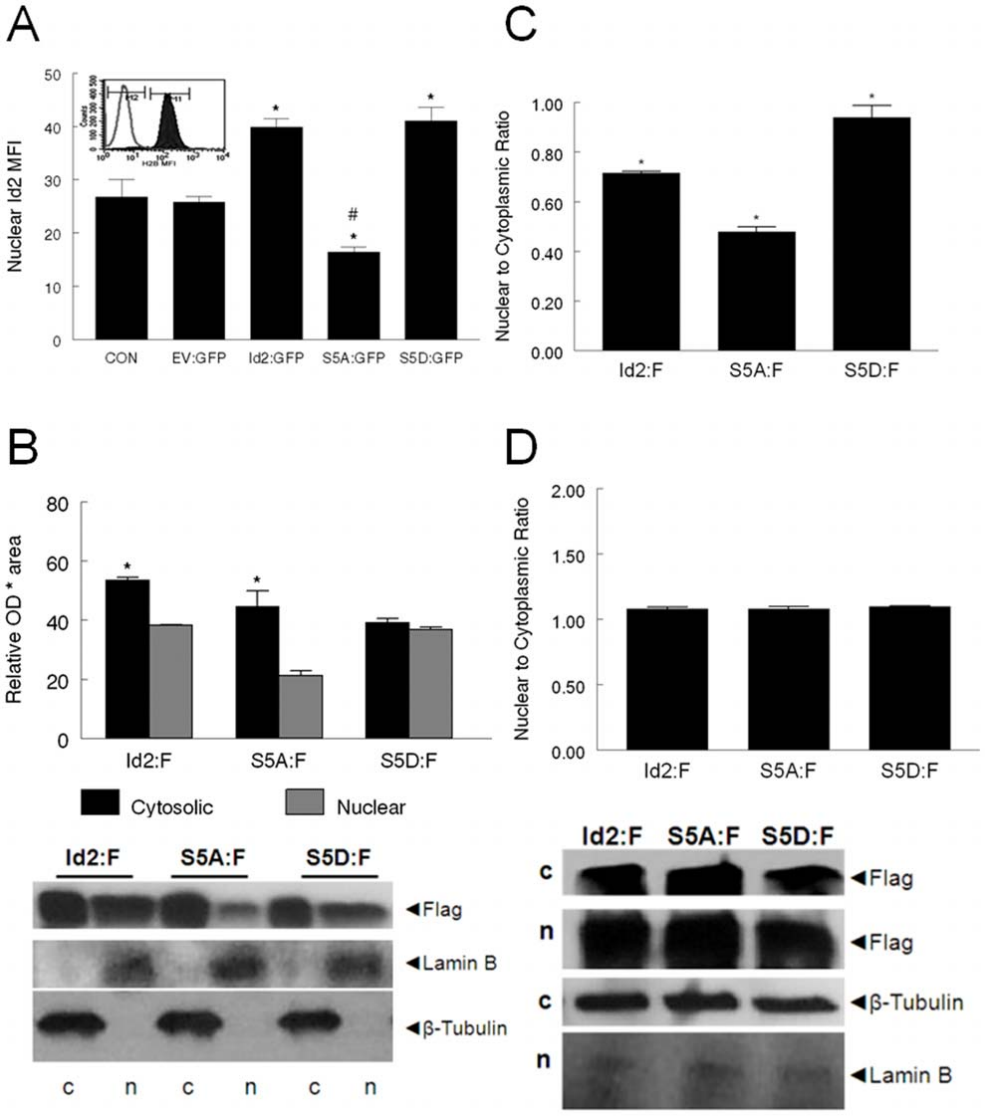
Subcellular localization of Id2. (A) Nuclear Id2 expression is decreased in myoblasts overexpressing S5A:GFP. C2C12 (CON) cells were transfected with, EV:GFP, Id2:GFP, S5A:GFP, and S5D:GFP. Twenty-four hours after transfection, the nuclei were isolated and subjected to flow cytometric analysis. [*, significantly different from CON, EV:GFP, and S5A:GFP; #, significantly different from all groups (p<0.05)] Insert: Histogram of Histone (H2B) expression (Comparisons of negative control □ and isolated nuclei ▪ *X-axis* = log of mean fluorescence intensity (MFI) of H2B. *Y-axis* = cell counts) (B) Nuclear flag expression is decreased in S5A:F transfected myoblasts. Nuclear (n) and cytoplasmic (c) Flag protein content was determined by Western blot analysis. Data are expressed as optical density (OD)×resulting band area expressed in arbitrary units×10^7^. Insert: representative blot for flag in Id2:F, S5A:F, and S5D:F transfected myoblasts. Controls for nuclear and cytoplasmic proteins were Lamin B and β-tubulin respectively. Data are means±SE. *, significantly different from Nuclear flag. #, S5:F significantly different from all groups (p<0.05). (C) The nuclear to cytoplasmic ratio of S5A:F is decreased in S5A:F transfected myoblasts. * #, significantly different from all groups (p<0.05). (D) LMB treatment causes Id2 to accumulate in the nucleus. Twenty-four hours following transfection, cells were treated with LMB (5 ng/ml) for 60 min, and separated into nuclear (n) and cytoplasmic (c) protein fractions as previously described.

To determine the subcellular localization of Id2, nuclei were isolated from C2C12 myoblasts transfected with EV:GFP, Id2:GFP, S5A:GFP, and S5D:GFP (Figure 4A). There was a significant increase in Id2 MFI in Id2:GFP and S5D:GFP transfected cells over CON and EV:GFP control myoblasts; however, there was a significant decrease in nuclear Id2 MFI in S5A:GFP transfected myoblasts (Figure 4A). To confirm these results, myoblasts were transfected with EV:F and Id2-3XFlag fusion vectors and separated into nuclear and cytoplasmic fractions (Figure 4B). Nuclear Id2 expression was significantly greater in Id2:F and S5D:F transfected myoblasts compared to S5A:F. The nuclear to cytoplasmic ratio Id2 was significantly lower in Id2:F and S5A:F groups compared to an almost 1∶1 ratio in phospho-mimicking S5D groups (Figure 4C).

Although all forms of Id2 were present in both nuclear and cytoplasmic protein fractions, phospho-ablated Id2 accumulated in the cytoplasm. It has been shown that Id2 is actively removed from the nucleus via a nuclear export receptor chromosome region maintenance protein 1 (CRM1) dependent mechanism. [30] To determine if the phosphorylation status of Id2 alters its nuclear accumulation, myoblasts were treated with a CRM1 specific nuclear export channel blocker Leptomycin B (LMB). Treatment of myoblasts with LMB (Figure 4D) resulted in an accumulation of Id2:F, S5A:F, and S5D:F in the nuclear fraction. The initial time-course experiments showed that prolonged exposure (3 hr) to LMB resulted in significant cell death based on morphological analysis (data not shown). As a result, these data were confirmed by immunocytochemistry (Supplement Figure S2). These data suggest that Id2 localizes to the nucleus, but phosphorylated Id2 may be retained in the nucleus. In contrast, phospho-ablated Id2, and possibly unphosphorylated Id2 may be exported to the cytoplasm under normal conditions when nuclear export is not inhibited.

### Phospho-ablated Id2 is pro-apoptotic in C2C12 myotubes

**Figure 5:**
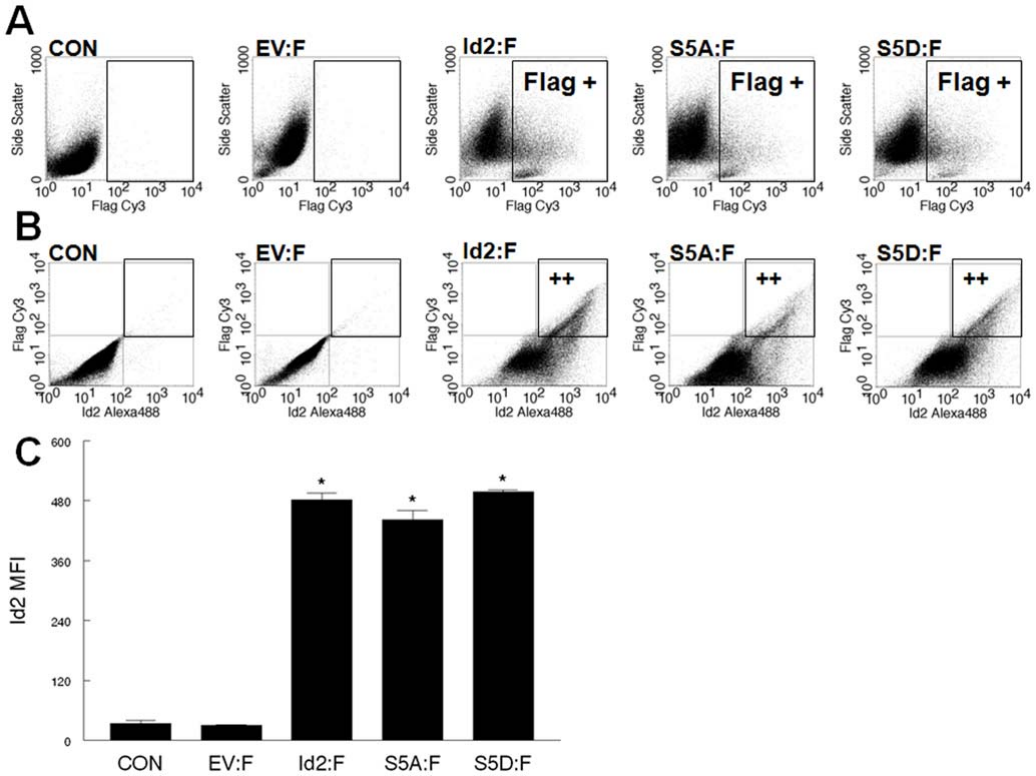
Phospho-ablated Id2 is pro-apoptotic in C2C12 myotubes. C2C12 myoblasts were transfected with, EV:F, Id2:F, S5A:F, and S5D:F. Twenty-four hours after transfection, cells were placed in DM and myotubes were harvested 72 hrs later. (A) Immunohistochemical staining of myotubes (MF20: Red, DAPI: Blue). (B) Myotube fusion index is the number of nuclei within a myotube divided by the total number of nuclei. As expected, the overexpression of Id2 delays myotube formation. [* significantly different from control EV:F (p<0.05): #, significantly different from EV:F and Id2:F (p<0.05)]. (C) Representative blots for MF20, β-tubulin, and Flag in transfected myotubes. (D) Caspase 3 activity assay. [*, Significantly different from all groups (p<0.05)].

Muscle growth and repair from injury are dependent on the ability of muscle precursor cells to proliferate, differentiate, and fuse into myotubes, and eventually, myofibers. As a result, apoptosis of myoblasts should inhibit myogenesis. The next experiment was designed to determine if the phosphorylation status of Id2 affects myoblast differentiation. C2C12 myoblasts were transfected with EV:F, Id2:F, S5A:F, and S5D:F. Twenty-four hours after transfection, the myoblasts were induced to differentiate by switching from growth medium (GM) to differentiation medium (DM). After 72 h in DM, MF20 immunostaining was performed, and the proportion of myosin heavy chain (MHC)-positive cells and the fusion index was measured as an indication of differentiation. The overexpression of Id2:F, S5A:F, and S5D:F significantly decreased the extent of myotube formation compared to control cells (Figure 5A & Figure 5B). The myotube index was not different between phospho-ablated Id2S5A and phospho-mimicking Id2S5D; however, there was a significant (p<0.05) reduction in total DAPI labeled nuclei (Figure 5A), as well as MF20 protein levels (Figure 5C) in Id2S5A transfected cells. Pro-apoptotic caspase 3 activity levels were also significantly elevated in S5A:F transfected myotubes. Together these data suggest that fewer myotubes survived in the Id2S5A transfected group, and they were likely eliminated via apoptotic regulated pathways. Nevertheless, the Id2S5A-transfected cells that remained in the dish appeared to differentiate normally.

## Discussion

A fraction of muscle precursor cells/myoblasts undergo apoptosis *in vivo* during muscle growth and repair from injury [31]–[33]; however, the mechanism regulating apoptosis during myogenesis especially in mature non-diseased muscles is not clearly understood. Previous research in our lab has shown a positive correlation between Id2 protein levels and apoptotic markers in aged skeletal muscle [22], as well as apoptosis induced atrophy of skeletal muscles [34]. In the present study, we have provided novel evidence that the phosphorylation status of Id2 is a mediator of apoptosis in C2C12 myoblasts. Specifically, the ectopic expression of phospho-ablated Id2 induced apoptosis in proliferating C2C12 myoblasts. Furthermore, we confirmed that Id2 overexpression reduces myogenic differentiation.

### Phospho-ablated Id2 is growth inhibitory in C2C12 myoblasts

Skeletal muscle growth and regeneration/repair are critically dependent on having an adequate pool of muscle precursor cells or myoblasts. The identification and characterization of important proteins that regulate the expansion and survival of myoblasts are important for both increasing our understanding of myogenesis, but also for identifying strategies that will reduce the loss of muscle mass that occurs with aging and disease. Enhanced myoblast proliferation could expand the number of myonuclei available for myotube formation. Id2 contains a consensus phosphorylation site (SPVR) for cyclin E/cdk2 and cyclin A/cdk2. Id2 is phosphorylated by cyclin E/cdk2 complexes during late G1, which results in a restoration of E-box transcriptional activity [15]. Our data suggest that phosphorylation of Id2 at serine-5 has an important role in regulation of myoblast proliferation.

In the current study, we found that the overexpression of Id2 increased the proliferation of C2C12 myoblasts. Conversely, we found that phospho-ablated Id2 transfected myoblasts had a significant decrease in S-phase entry. We speculate that the proliferation capabilities of Id2 and S5A occur in part through inhibition of p21 promoter activity. This hypothesis is consistent with previous observations showing that the promoter activity of p21 in rat aortic smooth muscle is not inhibited by phospho-ablated Id2 [16]. We found that p21 promoter activity was significantly decreased in myoblasts overexpressing Id2, and increased in myoblasts overexpressing phospho-ablated Id2S5A (Supplement Figure S3). Cyclin A is important for DNA replication and progression through S phase, and it requires cdk2 to enter the nucleus during prophase [35]. When Id2 is overexpressed in A549 carcinoma cells, there is a decrease in cyclin A promoter activity compared to control cells [13]. A decrease of cyclin A protein levels in myoblasts would be expected to decrease their proliferative capabilities. The possibility exists that the phosphorylation status of Id2 may alter cell cycle progression by regulating cyclin A expression. A prerequisite for myotube formation is p21 expression and exit from the cell cycle. This study is the first demonstration that Id2 phosphorylation status determines the proliferative status of skeletal muscle myoblasts. Similar results have been demonstrated in NIH3T3 cells, U2OS cells, and rat aortic smooth muscle cells [15], [16]. It is possible that the localization of Id2 may also be important in regulating passage through S phase.

### Phosphorylation and cellular localization of Id2

Id2 is primarily localized in the cytoplasm in skeletal muscle tissue that is undergoing apoptosis-associated atrophy [26], [27], [36]. Our results suggest that overexpression of unphosphorylated Id2 in proliferating myoblasts is chiefly restricted to the cytosol, where it may exert an apoptotic effect. A similar result was found in rat aortic smooth muscle cells [16]. However, because Id2 is a small protein, it can passively diffuse into the nucleus [30]. The C-terminal region of Id2 contains a nuclear export signal, and deletion of amino acids 103–109 result in nuclear localization [30]. Treatment of Id2 transfected NIH3T3 fibroblasts with a nuclear export inhibitor LBM, was previously shown to result in an accumulation of Id2 in the nucleus [30]. Interestingly, the cytoplasmic localization of Id2 decreases its ability to repress bHLH transcriptional activation of E-box promoter [30]. The ectopic expression of E-proteins significantly upregulates cyclin dependent kinase inhibitors (CDKI) p21, p15 and p16 and increases cell death via apoptosis [37].

### Phospho-ablated Id2 regulates apoptosis

Id2 has been shown to promote apoptosis in various cell types [8]. The N-terminal region of Id2, which contains the consensus cdk2 phosphorylation sequence SPVR, [15] has been shown to promote apoptosis in 32D.3 myeloid cells through an association with pro-apoptotic protein Bax [21]. During unloading-induced muscle atrophy after hypertrophy in young adult birds, there is a positive correlation between cytosolic Id2 protein levels and apoptotic markers Bax and TUNEL. Conversely, there is a negative correlation with the anti-apoptotic protein B-cell leukemia/lymphoma 2 (Bcl2) and Id2 [26]. In the current study, overexpression of phospho-ablated Id2 resulted in an increase in several markers of apoptosis including: Annexin V, TUNEL, caspase 3 and caspase 8 activities. Currently, the mechanism of how Id2 activates apoptosis remains unknown, but it may function through intrinsic (i.e., mitochondria) or extrinsic (i.e., death receptor) pathways. Cytochrome c release has been shown to activate processing of procaspase 8 into its active form [38], [39]. The possibility exists that phospho-ablated Id2 interacts with the death domain cascade, as caspase 8 has been shown to activate caspase 3 in MDA-MB231 breast cancer cells treated with etoposide [40]. Alternatively, the activation of caspase 8 that was seen in our results, could be due to the intracellular processing of procaspase 8 independent of the death receptor pathway, as caspase 8 has been shown to be activated by caspase 3 and caspase 6 in Jurkat cells [41]. In MCF-7 human breast cancer cells, caspase 8 activation appears to be due to the activation of caspase 9 within the apoptosome, as caspase 8 processing is inhibited by overexpression of the anti-apoptotic protein Bcl2 [42].

### Id2 phosphorylation status does not affect E47/E12 binding

Previous research has shown that Id2 preferentially forms heterodimers with class A bHLH proteins E47/E12, which are ubiquitously expressed throughout many different cell types [43]. MyoD, E12, and E47 are synthesized in the cytoplasm and translocated into the nucleus via a nuclear localization signal (NLS). Our results demonstrate that wild-type Id2, phospho-ablated Id2 and phospho-mimicking Id2 successfully bind to E47/E12 in C2C12 myoblasts. This suggests that Id2 binding to E proteins is not affected by the phosphorylation status of Id2. However, this does not rule out the possibility that Id2 phosphorylation might alter Id2:E protein function. For example, the phosphorylation of Id2 at serine 5 has been shown to abolish Id2's inhibition of E12/MyoD/DNA complexes *in vitro* [15]. It is likely that the apoptosis seen with phospho-ablated Id2 in C2C12 cells is not related to MyoD or bHLH proteins because, in 32d.3 myeloid cells, the overexpression of Id2 promotes apoptosis in a dose dependent manner independent of bHLH binding [21]. Deletion of the HLH domain renders Id2 less stable, but increases the amount of apoptosis [21]. In contrast, N-terminal deletions exhibit no changes in amount of apoptosis compared to wild-type Id2 transfectants based on Tunnel labeling and Bax expression and an increased percentage of sub-diploid DNA content [21]. Our data are consistent with previous observations showing that the phosphorylation of Id2 by protein kinase A and protein kinase C does not alter Id2:E47/E12 heterodimers or inhibition of DNA binding [44].

### The phosphorylation status of Id2 at serine 5 alters myotube formation and apoptosis

Id2 is a HLH protein that negatively regulates cell differentiation in many cell types including skeletal muscle [8]–[11]. Id proteins share a sequence homology with the second amphipathic helix by other basic HLH (bHLH) proteins such as MyoD, E47, and E12 [12]. The subcellular localization of MyoD and E47 are predominantly nuclear despite their dimerization partner [45]. Id proteins lack the basic N-terminal region needed for DNA binding and inhibit myogenic differentiation by sequestering bHLH proteins E47 and E12. Consistent with this role for Id2, we observed that in proliferating myoblasts there is no detectible binding of MyoD to the E47/E12. MyoD levels remained constant even with the overexpression of Id2, phospho-ablated Id2S5A, and phospho-mimicking Id2S5D. The cytosolic content of Id2 is increased and is also positively correlated to apoptosis in rodent and bird models of unloading-induced atrophy [26], [27]. Both mitotic and post-mitotic myonuclei are eliminated through apoptosis-induced mechanisms in unloading-induced atrophy [46]. Myotube formation was significantly reduced in the current study by the overexpression of Id2, Id2S5A, and Id2S5D, and there was a significant increase of caspase 3 activities in myotubes overexpressing phospho-ablated Id2. Furthermore, there was a decrease in the total number of nuclei in the myotubes overexpressing Id2S5A. The reduction of myotube formation is due to the combined effects of a decrease in proliferation and enhanced apoptosis. In human diploid fibroblasts, the phosphorylation of Id2 at serine 5 has been shown to abolish Id2's inhibition of E12/MyoD/DNA complexes *in vitro* [15]. In the present study, myotubes that overexpressed phospho-mimicking Id2S5D had a large number of unfused myonuclei as well as single cells expressing MF20. Previously, Id2 has only been correlated to apoptosis in skeletal muscle, and this is the first example of Id2 inducing apoptosis in myotubes. Future studies are needed to determine the mechanism of how Id2 phosphorylation at serine 5 alters myotube formation, and to determine if Id2 induces apoptosis in vivo.

In conclusion, our current data provide an explanation for previous observations indicating that cytoplasmic levels of Id2 and nuclear apoptosis are increased under conditions of muscle wasting including sarcopenia [26], [27]. In the present study, we have provided novel evidence that the phosphorylation status of Id2 is a mediator of apoptosis and cellular proliferation in C2C12 myoblasts. Specifically, the ectopic expression of phospho-ablated Id2 induced decreased proliferation of C2C12 myoblasts, and induced apoptosis. It is possible that muscle wasting increases hypophosphorylated levels of Id2, which are confined to the cytoplasmic spaces of muscle cells, and this initiates the increased apoptosis seen under these conditions of muscle loss [26], [27]. Future studies are however, needed to determine if the phosphorylation status of Id2 at serine 5 alters apoptotic markers seen during conditions of *in vivo* skeletal muscle loss including aging and disuse-induced muscle wasting. This is important because inhibition of apoptosis is likely to improve the number of precursor cells [3] that are available to participate in regeneration and repair of muscles following injury, or to improve muscle recovery following aging-associated losses.

## Materials and Methods

### Plasmid constructs

For construction of the wild-type Id2 plasmids, cDNAs encoding full-length murine Id2 were inserted into the Nde1/H*ind*III sites of the pDNR-1r vector (BD Biosciences, Palo Alto, CA, USA). Complementary DNA (cDNA) was reverse transcribed from RNA containing high levels of the Id2 transcript [47]. The forward primer (5′tccctcccggcctttcctccta3′) and the reverse primer (5′ccggagacacctggggagatgatc3′) generated a 650 bp product, which was used as a cDNA template for a second set of Id2 primers producing a 434-bp product. The forward primer (5′cctacgagccatatgaaagccttcag3′) contained an NdeI restriction site. The reverse primer (5′tccccaaataagcttttattagccacagagtactt3′) contained a hind III restriction site. The 434-bp Id2-PCR product was sequenced (SeqWright, Houston, TX, USA), and then subcloned into the pDNR-1r vector. The Id2-pDNR-1r vector was recombined with a pLP-IRES-eGFP vector (BD Biosciences) to generate an enhanced green fluorescent protein (GFP) expression vector (Id2:GFP) 6807-bp. For control experiments, the pDNR-1r vector was recombined with the pLP-IRES-eGFP vector to generate an empty GFP expression vector (EV:GFP). The IRES sequence in the Id2:GFP and EV:GFP vectors permitted both the wild-type Id2 gene, and the GFP gene to be translated from a single bicistronic mRNA. The EV:GFP vector only produced GFP.

### Site directed mutagenesis

The Id2-pDNR-1r vector was mutated with the QuickChange Site-Directed Mutagenesis kit (Stratagene, La Jolla, CA, USA) as suggested by the manufacturer. Phospho-ablated Id2 mutants (S5A) and phospho-mimicking Id2 mutants (S5D) were generated by changing serine 5 to alanine 5 and aspartic acid 5, respectively. The S5A forward and reverse mutant primers were 5′gaccatatgaaagccttcgcaccggtgaggt ccgtg3′ (mutation site is underlined), and 5′cctaacggacctcaccggtgcgaaggcttycatatc3′, respectively. The S5D forward and reverse primers were 5′gaccatatgaaagccttcgacccggtgaggtccgtg3′, and 5′cctaacggacctcaccgggtcgaaggctttca tatc3′, correspondingly. Id2, S5A, and S5D were cloned into pLP-IRES-eGFP as described above. C-terminal fusion proteins were also made by subcloning Id2, S5A, and S5D into p3XFlag-CMV-14 (Sigma-Aldrich, St. Louis, MO, USA) to yield Id2-flag (Id2:F), S5A-flag (S5A:F), and S5D-flag (S5D:F). An empty p3XFlag-CMV-14 (EV:F) vector was used as a control for flag experiments.

### Culture conditions for C2C12 myoblasts

C2C12 myoblasts (ATCC, Manassas, VA, USA) were maintained in growth medium (GM) consisting of Dulbecco's modified Eagle's medium supplemented with 10% FBS, 100 U/ml penicillin G, 100 µg/ml streptomycin, and 0.25 µg/ml amphotericin fungizone. The cells were incubated at 37°C in a water-saturated atmosphere of 95% ambient air and 5% CO_2_. Subsequent experiments were performed when the cells reached a density of approximately 70%.

### Electroporation-induced transfection of C2C12 myoblasts

C2C12 myoblasts were suspended in trypsin, centrifuged, and then resuspended in 100 µl of electroporation buffer (88 mM KH_2_PO_4_, 14 mM NaHCO_3_, 2.2 mM glucose, 14.6 mM ATP, and 23.5 mM MgCl_2_). Approximately 2×10^6^ cells per sample were electroporated with 4 µg of plasmid DNA, in a 0.2 cm gap cuvette (BioRad, Hercules, CA, USA), with four 200 V pulses each lasting 5 ms duration. After the electroporation, the cells were resuspended in 500 µl of minimal essential medium (ATCC, Manassas, VA, USA) for 5 min at 37°C. The cells were then plated into 10 cm plates with GM, and incubated in 5% CO_2_-95% air at 37°C, and then harvested 24 hrs later.

### Analysis by flow cytometry

Approximately 5×10^5^ C2C12 myoblasts per sample were used for antibody staining and cell cycle analysis. The adherent cells were harvested with trypsin, washed in 1× phosphate buffered saline (PBS), collected by centrifugation, and fixed with 4% paraformaldehyde. The cells were washed in PBS, and then incubated in permeabilization buffer (PB; 1× PBS containing 0.5% BSA, 0.025% TritonX), and collected by centrifugation. The cells were resuspended in PB containing an antibody to: Id2 (BD Biosciences, San Jose, CA USA), E47/E12, (BD Biosciences), MyoD (Santa Cruz, Santa Cruz, CA, USA), or M2 flag (Sigma-Aldrich) diluted at a final concentration of 1∶50 for 30 min at room temp. The cells were washed and incubated for 1 h in an appropriate secondary antibody [Alexa Fluor® 488, 546 goat anti-rabbit or mouse IgG, (Molecular Probes, Eugene, OR, USA); Cy5 Donkey anti-rabbit IgG (Jackson ImmunoResearch, West Grove, PA, USA); PE-Cy5 goat anti-mouse IgG1 (BD Biosciences)]. Negative controls were incubated without primary antibodies. After washing, the cells were collected by centrifugation, and resuspended in 400 µl of PB. Data acquisition and analysis were performed on a FACSCalibur™ Flow Cytometer using CellQuest Pro software (BD Biosciences). All data are represented as mean fluorescence intensity (MFI). Additional cell cycle modeling was performed using Modfit LT software (Verity Software House, Inc. ME, USA).

### TdT-mediated dUTP nick end labeling

DNA fragmentation of individual cells was detected in situ by a fluorescein labeled TdT-mediated dUTP nick end labeling (TUNEL) kit according to the manufactures directions (Roche, Philadelphia, PA, USA).

### Nuclear isolation

Adherent cells were harvested in trypsin, and washed twice in 1× PBS. The nuclei were isolated in 200 µl of Pipes-Triton Buffer (10 mM Pipes, 0.1 M NaCl, 2 mM MgCl_2_, and 0.1% Triton×100) per 2×10^5^ cells, and incubated for 30 min at 4°C. The nuclei were washed in PBS containing 1% BSA, centrifuged at 3000 rpm then stained with antibodies to flag-Cy3 or Id2, at a final concentration of 1∶50. The nuclei were washed in PB then incubated in PE-Cy5 goat anti-mouse IgG1 at a concentration of 1∶500. The nuclei were washed in PB, and resuspended in 500 µl of 1% PB containing 1 µg of propidium. Id2-Cy5 and Flag-Cy3 mean fluorescent intensity were measured by flow cytometry.

### Fluorometric caspase activity assay

Caspase activities were measured as previously reported [25], [48]. In brief, the total protein fraction (50 µl) without protease inhibitor of transfected C2C12 myoblasts was incubated in 50 µl of assay buffer (50 mM PIPES, 0.1 mM EDTA, 10% glycerol, and 10 mM DTT, pH 7.2) with 50 µM of the fluorogenic 7-amino-4-trifluoromethyl coumarin (AFC)-conjugated substrate (Ac-DEVD-AFC for caspase 3, IETD-AMC for caspase 8; Alexis, San Diego, CA) at 37°C for 2 h. The change in fluorescence was measured on a spectrofluorometer (CytoFluor; Applied Biosystems, Foster City, CA, USA) with an excitation wavelength of 390/20 nm and an emission wavelength of 530/25 nm before and after the 2-h incubation. Caspase activity was estimated as the change in arbitrary fluorescence units normalized to milligrams of protein used in the assay. Control and experimental samples were run on the same microplate in the same setting.

### Immunoprecipitation of Id2

Cells were harvested and homogenized in CHAPS buffer (40 mM HEPES (pH 7.5), 20 nM NaCl, 1 mM EDTA, 10 mM pyrophosphate, 10 mM β-glycerolphosphate, 40 mM NaF, 1.5 mM sodium vanadate, 0.3% CHAPS, 0.1 mM PMSF, 1 mM benzamidine, and 1 mM DTT). The resulting homogenate was centrifuged at 1000 g to obtain the supernatant. 500 µg of supernatant protein was combined with 5.8 µl of anti-flag M2 antibody and mixed overnight at 4°C. The immune complexes were isolated using a goat anti-mouse BioMag IgG (PerSeptive Diagnostics, Cambridge, MA, USA) bead slurry. The beads were blocked with 0.1% nonfat dry milk in CHAPS buffer, washed in CHAPS buffer, and then incubated with the sample for 1 hr at 4°C. The beads were collected using a magnetic stand, washed in CHAPS buffer containing 120 mM NaCl and 40 mM HEPES, and then in CHAPS buffer containing 200 mM NaCl and 60 mM HEPES. The precipitates were eluted in sample Laemelli buffer containing sodium dodecyl sulfate (SDS) and then boiled for 5 min. The beads were pelleted by centrifugation; the supernatant was collected and subjected to sodium dodecyl sulfate polyacrylamide gel electrophoresis (SDS-PAGE). The proteins were transferred to nitrocellulose membranes, incubated in antibodies to flag M2, Id2, E47/E12, or MyoD. The resulting bands were visualized with ECL Advance (Amersham Biosciences, Piscataway, NJ, USA), and exposed to X-Ray film.

### Protein Isolation and Western Blots

C2C12 myoblasts were washed with PBS at room temperature. C2C12 cells were lysed at 4°C with 150 µl of 1× SDS sample buffer, then centrifuged at 10,000×g to obtain the total cellular lysate. The supernatant contained the total cell lysate. A RC/DC Protein Assay (BioRad) was used to determine protein concentrations according to manufactures directions. For cell fractionation experiments, cells were harvested according to methods described previously in our lab [25]–[27]. The protein concentration of each lysate was detected by a DC Protein Assay Kit (BioRad) according to manufacturer's directions. 40 µg of protein extracts were separated by 18% SDS-PAGE, transferred to a nitrocellulose membrane, and then probed with antibodies against Id2, M2 Flag, E47, or β-tubulin. ECL Advance (Amersham Biosciences) was used to detect the immunopositive bands by chemiluminescence.

### Immunohistochemistry

The cells were grown on glass coverslips, fixed for 1 hr in 2% paraformaldehyde, washed in PBS then permeabilized with 0.1% TritionX-100. The coverslips were incubated at 20°C for 1 hr in with an antibody to M2 flag (Sigma-Aldrich). The cells were washed and incubated for 1 hr in an Alexa Fluor® 546 goat anti-mouse IgG secondary antibody (Invitrogen, Carlsbad, CA, USA). The coverslips were washed and mounted in a medium containing 4′,6-diamidino-2-phenylindole (DAPI; Vector Laboratories, Burlingame, CA, USA) to indentify the nuclei.

### Statistical Analyses

Statistical analyses were performed using the SPSS 10 software package. One way analysis of variance (ANOVA) was used to compare differences in dependent variables from C2C12 (CON), EV:GFP, and Id2:GFP, S5A:GFP, S5D:GFP, EV:F, Id2:F, S5A:F, and S5D:F transfected myoblasts. A minimum of three separate trials were completed for each experiment. Statistical significance was accepted at P<0.05. All data are given as means±standard error of the mean (SE).

## Supporting Information

Figure S1 *Detection of Id2-3XFlag fusion proteins*. CON = control C2C12; EV = Flag empty vector; Id2 = Id2-Flag; S5A = Id2S5A-Flag; S5D = Id2S5D-Flag. (A) Y-axis = side scatter, x-axis = Flag MFI. (B) Double labeling of Id2 and flag. As expected, flag positive myoblasts are also Id2 positive. (y-axis = Flag MFI, x-axis = Id2 MFI) (C) Id2 MFI. The MFI of Id2 was significantly (p<0.05) elevated in Id2, S5A, and S5D transfected myoblasts compare to control samples.
(2.66 MB TIF)

Figure S2 *LMB treatment causes Id2 to accumulate in the nucleus*. Twenty-four hours following transfection, cells were treated with (+) LMB (5 ng/ml) or vehicle (−) for 60 min, and immunohistochemical staining of M2Flag was performed (Flag: Red, DAPI: Blue).
(2.56 MB TIF)

Figure S3 *p21promoter activity is decreased by Id2*. A p21p-pGL2-luciferase reporter plasmid containing the promoter of human p21/WAF1 between positions −2,300 and +8 or pGL2-empty vector was cotransfected with CON, EV:F, Id2:F, S5A:F, and S5D:F. Twenty-four hours after transfection, a commercially available luciferase assay system from Promega was used to assess luciferase activity. Cells were lysed in passive lysis buffer as supplied by the manufacturer, and 20 µg of the cell lysate was assayed for luciferase activity using a standard luminometer. [* significantly different from control CON, EV:F and S5A:F (p<0.05 )]
(1.41 MB TIF)
