# Supplementary figures and images for: Phospho-Ablated Id2 Is Growth Suppressive and Pro-Apoptotic in Proliferating Myoblasts
Source: PLoS One. 2009 Jul 17;4(7):e6302. doi: 10.1371/journal.pone.0006302 (PMC2706990; doi:10.1371/journal.pone.0006302)

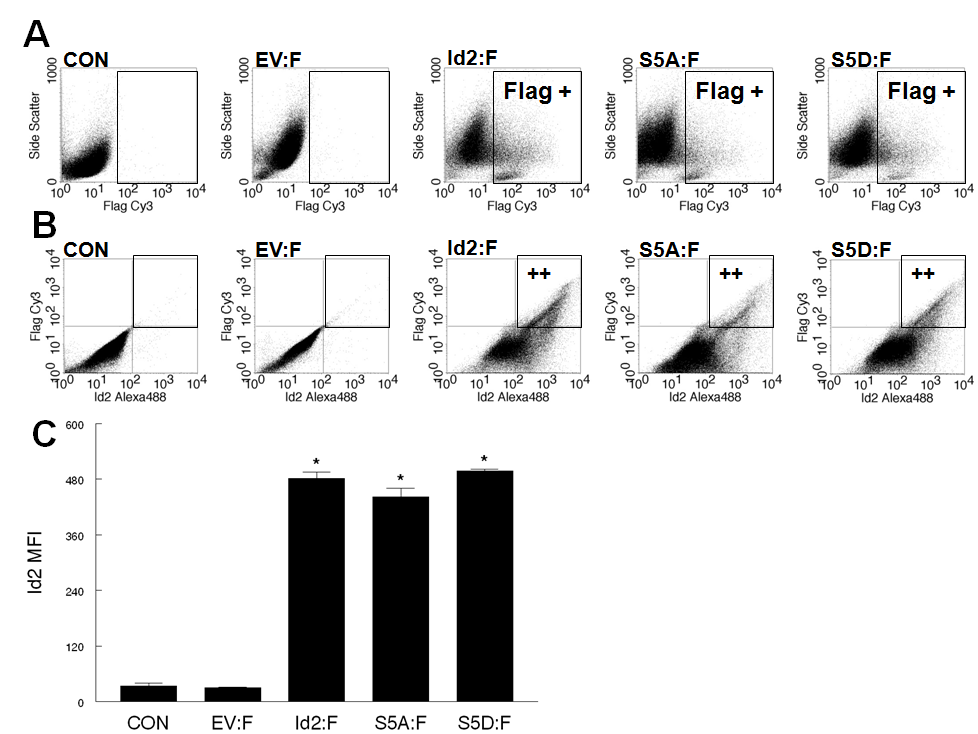

Supplement: Figure S1 — Detection of Id2-3XFlag fusion proteins. CON = control C2C12; EV = Flag empty vector; Id2 = Id2-Flag; S5A = Id2S5A-Flag; S5D = Id2S5D-Flag. (A) Y-axis = side scatter, x-axis = Flag MFI. (B) Double labeling of Id2 and flag. As expected, flag positive myoblasts are also Id2 positive. (y-axis = Flag MFI, x-axis = Id2 MFI) (C) Id2 MFI. The MFI of Id2 was significantly (p<0.05) elevated in Id2, S5A, and S5D transfected myoblasts compare to control samples. (2.66 MB TIF) [file pone.0006302.s001.tif]

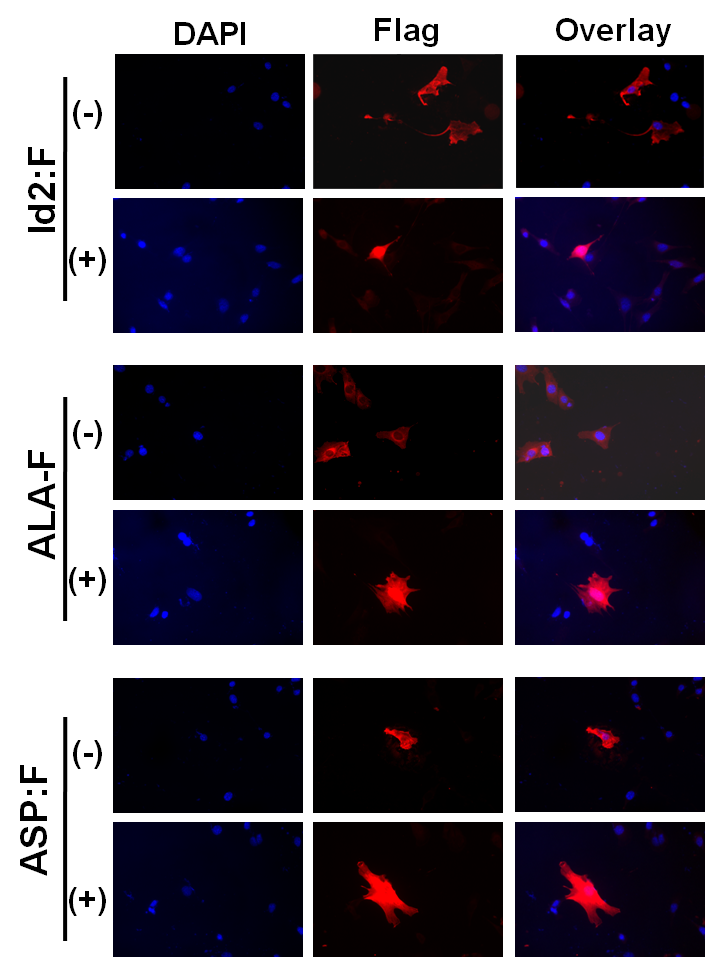

Supplement: Figure S2 — LMB treatment causes Id2 to accumulate in the nucleus. Twenty-four hours following transfection, cells were treated with (+) LMB (5 ng/ml) or vehicle (−) for 60 min, and immunohistochemical staining of M2Flag was performed (Flag: Red, DAPI: Blue). (2.56 MB TIF) [file pone.0006302.s002.tif]

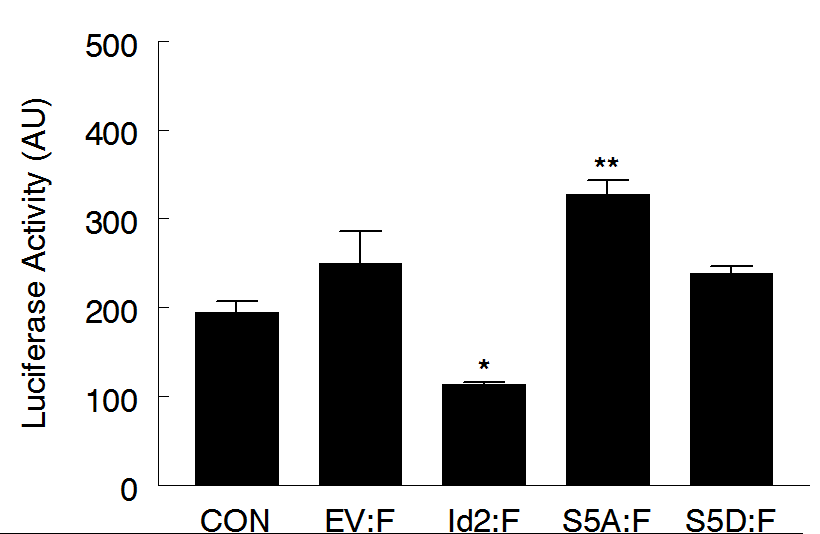

Supplement: Figure S3 — p21promoter activity is decreased by Id2. A p21p-pGL2-luciferase reporter plasmid containing the promoter of human p21/WAF1 between positions −2,300 and +8 or pGL2-empty vector was cotransfected with CON, EV:F, Id2:F, S5A:F, and S5D:F. Twenty-four hours after transfection, a commercially available luciferase assay system from Promega was used to assess luciferase activity. Cells were lysed in passive lysis buffer as supplied by the manufacturer, and 20 µg of the cell lysate was assayed for luciferase activity using a standard luminometer. [* significantly different from control CON, EV:F and S5A:F (p<0.05 )] (1.41 MB TIF) [file pone.0006302.s003.tif]
